# “Asymptomatic” Subclavian Steal Syndrome

**DOI:** 10.7759/cureus.19109

**Published:** 2021-10-28

**Authors:** Yu Amano, Takashi Watari

**Affiliations:** 1 Faculty of Medicine, Shimane University, Izumo, JPN; 2 General Medicine Center, Shimane University Hospital, Izumo, JPN

**Keywords:** brachial artery, blood pressure, syncope, subclavian artery, subclavian steal syndrome

## Abstract

The definition of subclavian steal is subclavian artery occlusion or hemodynamically significant stenosis proximal to the origin of the vertebral artery that results in lower pressure in the distal subclavian artery. Although subclavian steal can often remain asymptomatic, if ignored, it can cause syncope or neurological deficits. Detailed routine evaluation of blood pressure and careful physical examination, simultaneously on both sides of patients at high vascular risk (such as those with hypertension, dyslipidemia, and diabetes), can assist in the early detection. Herein, we report the case of an 82-year-old male patient with steal syndrome, who had no subjective symptoms despite severe stenosis of the subclavian artery, with a marked left-right difference in blood pressure.

## Introduction

The definition of subclavian steal is subclavian artery occlusion or hemodynamically significant stenosis proximal to the origin of the vertebral artery that results in lower pressure in the distal subclavian artery [1,2]. The subclavian steal syndrome is usually asymptomatic and is often diagnosed incidentally. Notably, it was first reported by Contorni in 1960 [3]. He was unable to palpate his patient’s radial artery, and subsequent angiography revealed stenosis of the artery. Similarly, Baltgaile et al. reported a case wherein the patient was completely asymptomatic despite having severe stenosis of the bilateral subclavian arteries [4]. Herein, we report the case of an 82-year-old male patient with steal syndrome who had a marked left-right difference in blood pressure; he had no subjective symptoms despite severe stenosis of the subclavian artery.

## Case presentation

An 82-year-old male patient presented with a marked left-right difference in blood pressure. The patient had a long-term history of hypertension, moderate chronic kidney failure, type 2 diabetes, and dyslipidemia. Additionally, he had been recording the blood pressure in his left arm daily at home. On inspection of his blood-pressure notebook during a medical consultation, we found that his left systolic blood pressure had dropped from around 130 mmHg to an average of 80 mmHg over a month. On re-testing in the examination room, the left systolic pressure was confirmed to be 80 mmHg, while his right systolic pressure was found to be approximately 150 mmHg. A detailed interview with the patient revealed no complaints of fever, numbness, dizziness, or pre-syncope. Physical examination of his left and right sides did not reveal visible evidence of pulsation of the left brachial artery (Video 1).

**Video 1:** Left-right difference in beating of brachial artery Left side (right arm), right side (left arm)

**Figure 1:**
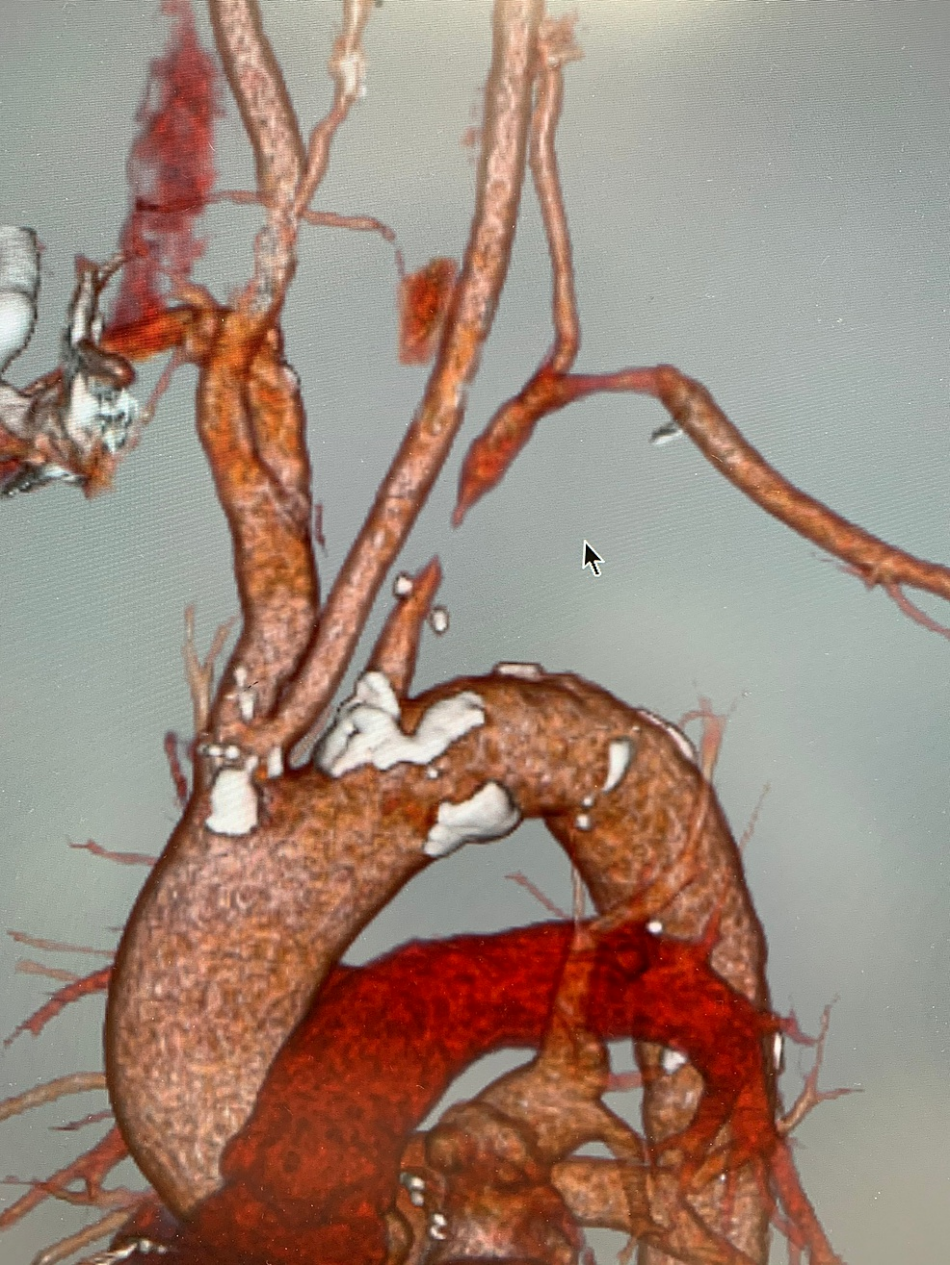
Contrast CT CT image revealed almost complete occlusion at the origin of the subclavian artery CT, computed tomography

Furthermore, there were apparent radial-artery pulse differences and slight temperature differences between the left and right sides. However, his hands did not show any observable differences in terms of the color tone. Moreover, there was no audible ejection murmur near the left subclavian artery. Due to the lack of subjective symptoms, we suspected asymptomatic subclavian steal syndrome despite left-right differences in the blood pressure. This was confirmed after we conducted contrast computed tomography, which revealed subtotal occlusion of the origin of the left subclavian artery and severe stenosis of the middle portion of the left vertebral artery (Figure 1). 

The patient was referred to a cardiac surgeon who prescribed low-dose aspirin for follow-up in six months.

## Discussion

The accurate prevalence of subclavian steal syndrome has not been established. Approximately 30% of patients who have been confirmed with a peripheral arterial disease have stenosis of the subclavian artery, but only a tiny percentage become symptomatic, primarily because of the widespread collateralization of vessels [5]. This is a case of an asymptomatic patient with subtotal occlusion of the subclavian artery and stenosis of the vertebral artery. In this case, a cardiac surgeon prescribed aspirin for follow-up, but the effect of an anticoagulant on the asymptomatic subclavian syndrome is unclear. However, this may be a reasonable treatment for such a patient, who is likely to have systemic vascular stenoses [5]. According to Tan et al., subclavian steal syndrome is often a differential diagnosis in any patient who presents with a pulse deficit or a systolic blood pressure difference greater than 20 mmHg between the left and right arms [6]. Labropoulos et al. and Osiro and Zurada showed that differences of 40 mmHg or more, between left and right-side blood pressure, were likely to trigger symptoms [7,8]. According to their data, 35%-40% of patients with a left-right blood pressure difference of more than 50 mmHg experience symptoms. However, in our case, the patient was asymptomatic despite a difference of approximately 70 mmHg and severe stenosis of both the subclavian and vertebral arteries. Through a detailed physical examination of the pulse simultaneously on both sides, we detected differences in radial pulse and temperature between the left and right sides. Therefore, detailed routine evaluation of blood pressure and physical examination simultaneously on both sides of patients at high vascular risk (such as those with hypertension, dyslipidemia, and diabetes) can assist in the early detection of severe vascular stenosis and associated diseases. 

## Conclusions

Subclavian steal can often remain asymptomatic. However, if ignored, it can cause syncope or neurological deficits. Detailed routine physical examination and evaluation of the blood pressure on both sides of patients at high vascular risk (such as those with hypertension, dyslipidemia, and diabetes) can assist in early detection and management.
